# *Haemonchus contortus* HcL6 promoted the Th9 immune response in goat PBMCs by activating the STAT6/PU.1/NF-κB pathway

**DOI:** 10.1186/s13567-023-01214-5

**Published:** 2023-09-22

**Authors:** Meng Liang, Yang Zhang, Mingyue Wang, Zhaohai Wen, Cheng Chen, Yongqian Bu, Mingmin Lu, Xiaokai Song, Lixin Xu, Xiangrui Li, Ruofeng Yan

**Affiliations:** 1 MOE Joint International Research Laboratory of Animal Health and Food Safety, College of Veterinary Medicine, Nanjing Agricultural University, Nanjing, 210095 Jiangsu China; 2 Jiangsu Vocational College of Agriculture and Forestry, Zhenjiang, 212400 Jiangsu China

**Keywords:** *Haemonchus contortus*, HcL6, goat, Th9 immunity, STAT6, PU.1, NF-κB

## Abstract

Th9 cells play a crucial role in parasite immunity. The development of Th9 cells is facilitated by several cytokines. Key transcription factors, such as STAT6, STAT5, and PU.1, are known to enhance IL-9 expression during the Th9 immune response. NF-κB-mediated transduction pathways participate in the induction of IL-9. In a previous study, we unveiled a unique ribosomal protein derived from *Haemonchus contortus* excretory-secretory proteins (HcESPs) that interact with host Th9 cells. In the present study, the effects of the *Haemonchus contortus* ribosomal protein L6 domain DE-containing protein (HcL6) on IL-9 secretion, Th9 differentiation, and IL-9 transcription were assessed by employing ELISA, flow cytometry, and qPCR methodologies. The observations revealed the transcriptional upregulation of several key genes within the Th9 immune response pathway. Moreover, silencing STAT6, PU.1, and NF-κB was found to attenuate the Th9 immune response. In this study, we unveiled the Th9 immune response-inducing capabilities of HcL6 and elucidated some of its underlying mechanisms. These findings suggest that HcL6 is an immunostimulatory antigen capable of inducing the Th9 immune response. These insights could prove instrumental in identifying potential candidate antigens for the development of immunoprophylactic strategies against *H. contortus* infections.

**Supplementary Information:**

The online version contains supplementary material available at 10.1186/s13567-023-01214-5.

## Introduction

IL-9 is a typical cytokine produced by Th9 cells. Several studies report that IL-9 and Th9 immune responses play protective and central roles in parasitic nematode infections [1–4]. However, the exact molecules that induce the Th9 immune response in *H. contortus* excretory-secretory proteins (ESPs) have not been elucidated.

A cytokine pool in the culture conditions is necessary to differentiate Th9 cells. The main cytokines that promote Th9 cell differentiation are IL-4 and TGF-β, as indicated in our previous study [5]. The transcript levels of the *IRF-4*, *PU.1*, *TGF-β1* and *Smad3* genes implicated in the TGF-β/Smad signaling pathway were significantly increased when the concentration of ESPs was greater than 10 μg/mL compared with levels in the control group. Th9 cell development requires transcription factors downstream of IL-4, including STAT6, IRF4, and GATA3 [6–9]. The TGF-β signaling pathway in Th9 cells is dependent on Smad activation and PU.1 expression [7, 10]. Furthermore, the development of the Th9 subset population is stimulated by IL-2 and STAT5. NF-κB is an important inducer of IL-9 gene expression and is associated with the development and production of Th9 cells [11, 12]. T cells induced IL-9 production from NFATc2 through translocation of calcitonin gene-related peptide (CGRP) [13]. CGRP promotes IL-9 secretion in a cAMP/PKA-dependent manner and upregulates GATA3 and Sfpi1 expression.

Ribosomal proteins perform other functions independent of the translation machinery in addition to their role in ribosome biogenesis. Previous findings have indicated that PbRPL6120-127, a putative 60S ribosomal protein L6 (RPL6) in *Plasmodium berghei* ANKA, protects the host against malaria by acting as an optimal antigen for generating hepatic TRM cells. In a single-dose vaccination targeting RPL6, sterilizing immunity was effective and prolonged against high-dose sporozoite challenges [14]. However, the role of ribosomal protein L6 in nematodes has not been reported in previous studies.

In a previous study, 218 *H. contortus* excretory-secretory proteins (HcESPs) interacting with goat Th9 cells were identified by liquid chromatography-tandem mass spectrometry (LC‒MS/MS) analysis. *H. contortus* ribosomal protein L6 domain DE-containing protein (HcL6) was detected among the proteins interacting with Th9 cells [15]. Research should be conducted to identify the molecules that directly or indirectly regulate Th9 cells at the parasite-host interface. HcL6 may be one of these dominant proteins that modulate the Th9 immune response owing to the functional diversity of the ribosomal L6 proteins. Therefore, the aim of this study was to characterize the functional properties of the HcL6 protein and to elucidate the relevant pathways involved in the regulation of the Th9 immune response.

## Materials and methods

### Ethical statement

The treatments of animals in our research conformed with the guidelines of the Animal Welfare Board of Nanjing Agricultural University, China. The protocols were approved by the Animal Ethics Committee of Nanjing Agricultural University, China. The Science and Technology Agency of Jiangsu Province authorized the trials (Approval ID: SYXK (SU) 2010–0005).

### Animals and cells

Wistar rats (female, body weight 180–220 g) were obtained from the Experimental Animal Center of Jiangsu, China (SCXK 2018–004), and were fed in the Animal Experimental Laboratory of Nanjing Agricultural University under aseptic conditions.

Five- to seven-month-old goats were reared in ventilated cages under clean and hygienic conditions to prevent accidental nematode infections. They were also provided with hay, water and alfalfa pellets at all times. During the study, no nematode eggs were found in the goat feces. As described previously, peripheral blood mononuclear cells (PBMCs) from goats were isolated and cultured using peripheral venous blood samples (10 mL per goat) obtained by venipuncture [16].

### Cloning of the gene encoding HcL6 and sequence analysis

Total RNA was extracted from *H. contortus* adult worms using TRIzol reagent (Invitrogen, New York, USA) [17]. The RNA (OD260/280 = 1.99, 1421.26 ng/μL) was transcribed by a cDNA Synthesis Kit (TaKaRa Biotechnology, Dalian, China) according to the manufacturer’s instructions. The gene encoding HcL6 was amplified by reverse transcriptase PCR (RT‒PCR) using specific primers (Table 1) based on the sequence of HcL6 (EBI No. CDL93639.1). The PCR product was ligated into a prokaryotic expression vector (pET-28a ( +)) (Novagen, USA) to generate recombinant HcL6, followed by endonuclease cleavage and sequence analysis (SnapGene 4.3.7, USA; Additional file 1).

**Table 1:** **Oligonucleotide sequences of primers used for HcL6 amplification**

Gene name	Abbreviation	Protein ID	Specific primers	Enzyme
Ribosomal protein L6 domain DE-containing protein	L6	W6NC67	CGCGGATCCATGAAGCTGGTCGAGTCC	*BamH* I
			CCCAAGCTTTCAGTCTTGAACAATAGTAGTTTTC	*Hind* III

### Expression and purification of recombinant HcL6 protein

The expression of recombinant HcL6 protein was performed as described [18]. In brief, the recombinant plasmid pET-28a-HcL6 was transfected into *E. coli* BL21 (DE3) and incubated in Luria-Bertini medium with kanamycin at 37 ℃ until the OD600 ranged from 0.6 to 0.8. Isopropyl-β-d-thiogalactopyranoside (IPTG) was added to induce protein expression. The His-tag-fused recombinant proteins were purified with a His Trap™ FF kit (GE Healthcare, USA) following the manufacturer’s protocol. A ToxinEraser™ Endotoxin Removal kit (Genscript Biotech Company, Nanjing, Jiangsu, China) was used to remove endotoxin from the sample. Afterwards, 0.22-µm filters were used to filter the rHcL6. A BCA method was used to determine the protein concentration (776.09 μg/mL) [19], and the size and purity of the rHcL6 protein were confirmed by 12% SDS‒PAGE gels.

### Preparation of polyclonal antibodies

Wistar rats were given 300 g of rHcL6 protein blended with Freund’s complete adjuvant (FCA, diluted 1:1, Sigma, USA) to generate polyclonal antibodies. After the primary immunization, the rats were boosted 4 times with the same dose of rHcL6 protein with Freund’s incomplete adjuvant (FIA, diluted 1:1, Sigma, USA) at one-week intervals. In rat sera, the specific IgG titer (1:2^23^) was determined by indirect enzyme-linked immunosorbent assay (ELISA) as previously described [20]. A negative control was collected from rats before vaccination. All sera were stored at −70 °C.

### Western blot analysis

rHcL6 proteins were resolved on 12% SDS‒PAGE gels and electrotransferred onto nitrocellulose membranes. The membranes were incubated in 5% skim milk in Tris-buffered saline containing 0.1% Tween-20 (TBST) at 37 ℃ for 1 h to block the nonspecific binding sites. The blots of rHcL6 samples were treated with rat anti-rHcL6 serum (1:300 in TBST) or normal rat serum (control) overnight at 4 ℃. After the membranes were washed five times in TBST, horseradish peroxidase (HRP)-conjugated rabbit anti-rat IgG secondary antibody (Sigma‒Aldrich) in TBST (1:5000) was incubated with the membranes for 1 h at 37 ℃. Finally, immunoreactions were detected by a DAB kit (Sigma‒Aldrich) with 3–5 min of colour development.

### Binding of HcL6 protein with Th9 cells in vitro

Total Th9 cells in goat PBMCs were sorted by the flow-sorting cytometry method as described elsewhere [15]. Briefly, PBMCs were cultured in RPMI-1640 media containing rHcL6 for 2 h at 37 °C in a humidified 5% CO_2_ incubator. Following that, phorbol myristate acetate (PMA, Sigma‒Aldrich, MO, USA) and ionomycin (Sigma‒Aldrich, MO, USA) were added to cells containing brefeldin A solution (BFA; BD Biosciences, San Jose, CA, USA) for another 4–6 h before intracellular dyeing was performed [5]. Then, the cells were obtained and incubated with fluorescein FITC-labelled anti-CD2, Alexa Fluor 488 dye-tagged CD4 antibodies (BD Biosciences, Becton, USA), PE-Cy5-conjugated anti-IL-9 (GenScript, New Jersey, USA) and PE-conjugated anti-IL-10 antibodies (BD, Pharmingen, USA). The labelled cells were suspended in 500 µL of PBS and analysed with BD FACS ARIA II SORP (BD Biosciences) [21]. The Th9 cell population was represented by the CD2 + CD4 + IL-9 + IL-10 + phenotype.

The collected Th9 cells were subjected to three washes using PBS (pH 7.4). Subsequently, Tide Quencher™ 3WS acid, Tide Quencher™ 2WS acid, and Tide Quencher™ 5WS acid (AAT Bioquest, CA, USA) were introduced to quench fluorescence. This process was conducted over 5 h until no residual fluorescence was detectable. After several additional washes, the cell suspensions were loaded onto adhesion microscope slides and allowed to settle for 15–20 min before the supernatant was discarded. The Th9 cells were fixed using 4% paraformaldehyde (PA) for 15 min at room temperature. This step was followed by incubation with 5% bovine serum albumin (BSA) in PBS, with added 0.1% Tween-20 (PBST), at 37 ℃ for 30 min. The slides were then incubated overnight at 4 ℃ with rat anti-rHcL6 IgG (1:300) or normal rat IgG (serving as a control). Following three washes with PBST, Cy3-labelled goat anti-rat IgG (1:500) was applied to the slides and incubated for 1 h at 37 °C. Three additional PBST washes preceded the nuclear staining with 2-(4-amidinophenyl)-6-indolecarbamidine dihydrochloride (DAPI, Sigma‒Aldrich) for 5 min at room temperature. An anti-fade medium (Sigma‒Aldrich) was utilized to prevent fluorescence quenching of the samples prior to microscopic examination. The final stage involved imaging the slides using an LSM780 laser scanning confocal microscope (Zeiss, Jena, Germany) at 100 × magnification. Digital images were processed and analysed using ZEN 2012 software (Zeiss).

### Detection of IL-9 transcription

The transcription of IL-9 was detected by real-time PCR. PBMCs (1 × 10^6^ cells/mL) were incubated with various concentrations of rHcL6 (5, 10, 20, 40, and 60 μg/mL) in vitro in 24-well culture plates supplied with RPMI-1640 medium for 24 h at 37 ℃. Subsequently, RNA was extracted from these cells using a Total RNA Kit (Omega, USA) according to the manufacturer’s instructions. The mRNA transcription of IL-9 was assessed as described elsewhere [15]. Specific primers for the β-actin gene (endogenous reference) and target gene IL-9 (F: 5′-GATGCGGCTGATTGTTT-3’; R: 5′-CTCGTGCTCACTGTGGAGT-3′) were used, and the relative transcription levels of IL-9 were normalized to β-actin based on the 2^−∆∆Ct^ method [5]. Real-time PCR was performed, and the data were recorded by an ABI 7500 instrument (Applied Biosystems, USA).

### IL-9 concentration assays

ELISA was used to examine the effects of rHcL6 on IL-9 secretion. PBMCs (1 × 10^6^ cells/mL) were stimulated with lipopolysaccharide (LPS, 100 ng/mL). Two hours following stimulation, 2 mL of cells (1 × 10^6^ cells/mL) was added to each well of a 6-well plate. Then, the cells were incubated with different concentrations of rHcL6 to determine whether the protein could stimulate IL-9 secretion. All the samples were incubated at 37 ℃ and 5% CO_2_. After 24 h of incubation, the supernatants were collected by centrifugation. The IL-9 concentration in the supernatants was measured by ELISA. Different concentrations of IL-9 protein (300 pg/mL, 150 pg/mL, 75 pg/mL, 37.5 pg/mL, 18.75 pg/mL, 9.38 pg/mL and 4.69 pg/mL) and 100 μL of each cell supernatant were placed into a 96-well plate containing IL-9 monoclonal antibody at 37 ℃ for 90 min. After removing the supernatant, rat anti-IL-9 IgG polyclonal antibodies coupled with biotin (1:200) were added to each well at 37 ℃ for 1 h. After being washed three times with TBST, the avidin–biotin-peroxidase complex was added at 37 ℃ for 30 min. After three washes with TBST, TMB was added for 15–20 min in the dark, and then ELISA stop solution was added to terminate the reaction. The optical density at 450 nm was measured using a microplate reader (Bio-Rad, Hercules, California, USA). Three independent experiments were performed in the test with three technical replicates for each group.

### Detection of Th9 cell differentiation

To estimate the effects of rHcL6 on Th9 cells, PBMCs were incubated with various concentrations of rHcL6 (5, 10, 20, 40, and 60 μg/mL) in vitro in 12-well culture plates supplied with RPMI-1640 medium for 12 h at 37 ℃ with 5% CO_2_. Therefore, the method is the same as that in the subsection “The binding of HcL6 protein with Th9 cells in vitro”.

### Detection of key genes in Th9 immune response signaling pathways

This study investigated the transcription of key genes in Th9 immune response signaling pathways, such as STAT1, STAT5, GATA3, SMAD, NFAT, IRF4, PU.1, STAT6 and NF-ĸB, by real-time PCR. PBMCs (1 × 10^6^ cells/mL) were incubated with 20 μg/mL rHcL6 in vitro in 24-well culture plates supplied with RPMI-1640 medium for 24 h at 37 ℃. Subsequently, the method was the same as that described in the subsection “Detection of IL-9 transcription”. The primers for these nine key genes are listed in Table 2.

**Table 2:** **Primer sequences for quantitative real-time PCR**

Target	Nucleotide sequence (5′–3′)	Efficiency	Tm (℃)	Amplicon size (bp)	Correlation coefficients (r^2^)
GATA3	AAACCCGATGGATCTGTG TGTGAGTCTGAATGGCTTATT	110%	51.26	141	0.9901
IRF4	TCGAGAAGGCATCGACAAGC GCTCTTGTTCAGAGCACACC	114%	56.14	70	0.9996
NFAT	CCCAGGAGAAACAGCATAAA TCTGATCCAGGGCGAGAC	118%	54.51	105	1
PU.1	GCAAGAAGAAGATCCGCCTGTACC TTGTGCTTGGACGAGAACTGGAAC	90%	60.05	121	0.9903
SMAD	TTTGTGGGTCGGATCATGGG CAGGGTCCACCGATTCCAAA	120%	56.01	150	1
STAT1	ATGCCACCGAACTTACCCAG AGCTGATCCAGGCAAGCATT	110%	56.52	112	0.9997
STAT6	AGAGGGAGACGACAACAGAG CAGTCACCCAGGAGATGC	91%	56.08	150	0.9995
STAT5	GGCTTTCTTTCTCATTTCC CAACACTCCACCCACCCT	101%	52.50	107	0.9901
NF-κB	CCACGTCACATCCAACCC GCTGGCATCAAGCGAAAA	102%	55.29	159	0.9979

### Knockdown of the STAT6/NF-κB/PU.1 gene by RNA interference

Three single sequence siRNA duplexes (RiBo Bio, Guangzhou, China) of STAT6/NF-ĸB/PU.1 siRNA were procured (Table 3). Transfection of these siRNAs was accomplished using RNAiMAX Transfection Reagent (Thermo Fisher, MA, USA). Following transfection, the culture medium was replaced with fresh medium, and after 48 h, the cells were ready for treatment. A dose‒response study was initially conducted to assess the impact of 0 nM, 10 nM, 20 nM, 40 nM, and 80 nM STAT6/NF-κB/PU.1 siRNA on the transcription level of STAT6/NF-κB/PU.1. Following this experiment, cells transfected with 20 nM STAT6, 20 nM NF-κB, and 40 nM PU.1 were used to examine the expression of IL-9 and key genes within the Th9 immune response signaling pathways.

**Table 3:** **Sequences of siRNAs for STAT6/NF-κB/PU.1**

Target	siRNA	siRNA sequence (5′–3′)
STAT6	siRNA-1	CTGGAGACCATATATCAGA
	siRNA-2	CAGGAGGAACTCAAGTTTA
	siRNA-3	GTACGTCACTAGCCTTCTT
NF-κB	siRNA-1	GGTCAAAGTGACTCCTGAT
	siRNA-2	GATCACAGTTCCACTGTCT
	siRNA-3	AGCTCATCGCTGGCAACAA
PU.1	siRNA-1	GCAAGAAGATGACCTACCA
	siRNA-2	CGCCAAACGCACGAATATT
	siRNA-3	CTCAGGATGGTTGACGCTT

### Statistical study

Statistical analyses were carried out using GraphPad Prism 7.0 (GraphPad Prism, USA). All data are presented as the mean ± SD. Differences between groups were evaluated by one-way ANOVA followed by Tukey’s post hoc test. The thresholds for statistical significance were set at *p < 0.05, **p < 0.01, and ***p < 0.001. Flow cytometry data were analysed employing FlowJo software (Version 10, USA).

## Results

### Cloning and expression of HcL6

**Figure 1:**
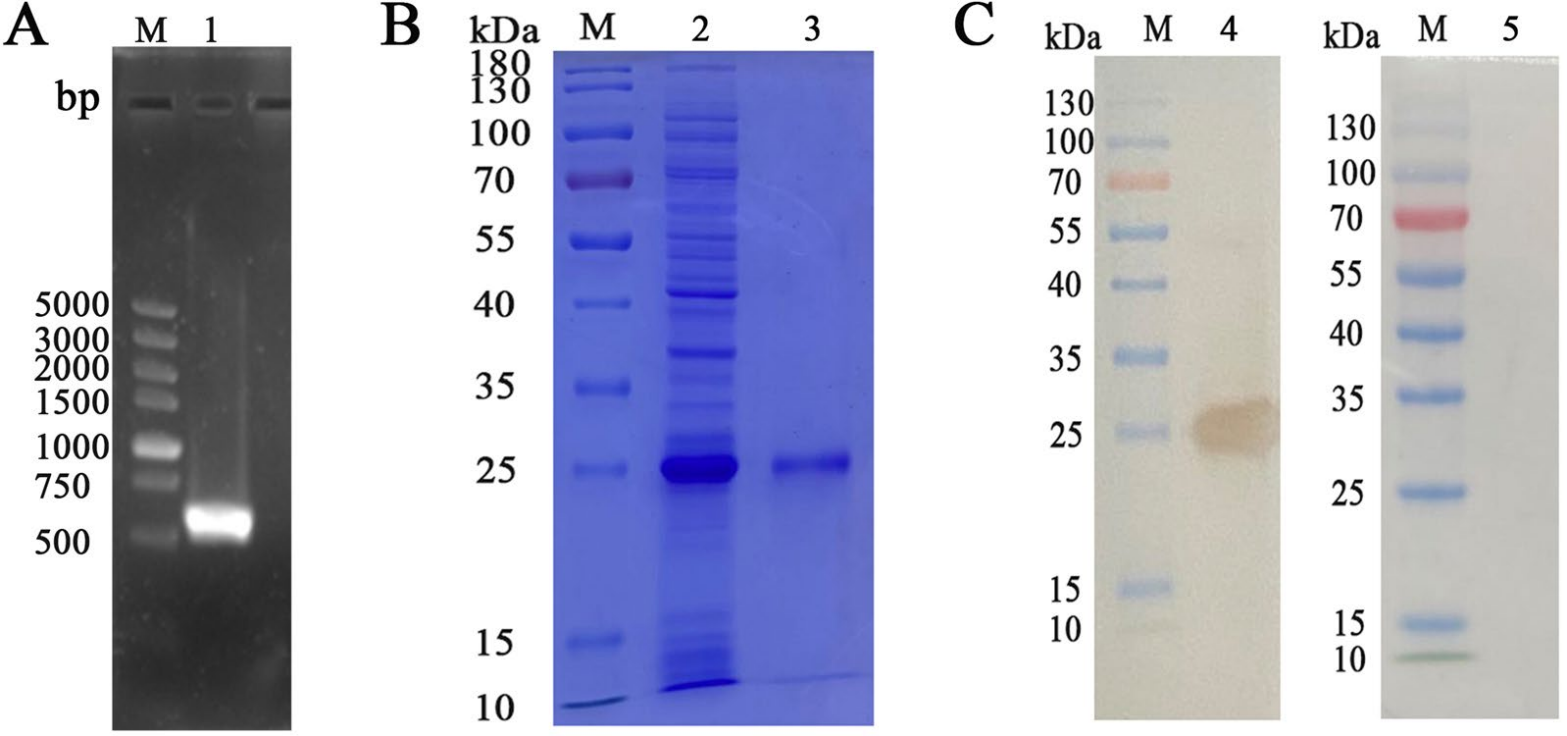
**Cloning and expression of HcL6**. **A**: Agarose gel electrophoresis of the HcL6 gene. M: DNA molecular weight marker; Lane 1: The amplification products of the HcL6 gene. **B**: Purification of rHcL6. Lane M: Standard protein molecular marker; Lane 2: rHcL6 expressed in the supernatant of cell lysates; Lane 3: purified rHcL6. **C**: Western blot. Lane 4: Immunoblot analysis of rHcL6 using rat anti-rHcL6 IgG as the primary antibody; Lane 5: Immunoblot analysis of rHcL6 using normal rat IgG (control) as the primary antibody.

The complete coding sequence of the HcL6 gene (567 bp), encoding a protein composed of 189 amino acids, was cloned from adult worms through polymerase chain reaction (PCR) (Figure 1A). This protein, with an anticipated molecular mass of approximately 24.79 kDa, was successfully expressed as a fusion protein with histidine tags in the cell lysate supernatant (Figure 1B, Lane 2). Upon purification, the rHcL6 protein was discerned to be a single band on SDS‒PAGE gels via Coomassie Blue staining, with a molecular weight of approximately 25 kDa (Figure 1B, Lane 3). Western blotting ascertained the specificity of the rHcL6 protein, as probing it with a rat anti-rHcL6 IgG yielded a single band of ~25 kDa (Figure 1C, Lane 4). In contrast, the control groups did not exhibit any positive bands (Figure 1C, Lane 5), demonstrating the specificity of the rat anti-HcL6 IgG against rHcL6.

### rHcL6 protein binding with goat Th9 cells

**Figure 2:**
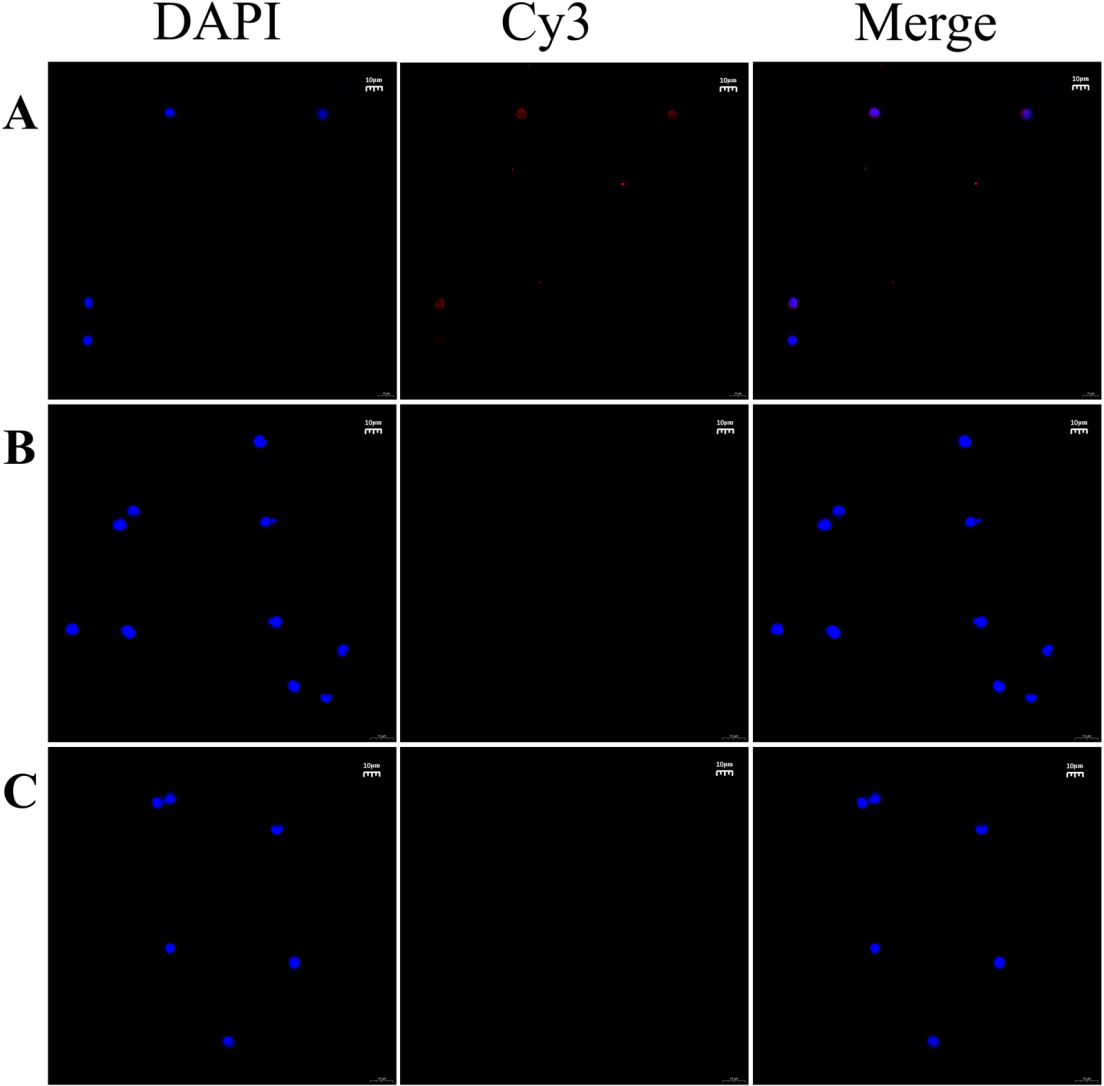
**rHcL6 binding with goat Th9 cells.** Goat PBMCs were incubated with rHcL6 for 2 h, and then Th9 cells were sorted by flow-sorting cytometry. Th9 cells treated with (**A**) or without (**C**) rHcL6 protein were incubated with rat anti-rHcL6 IgG as the primary antibody. Th9 cells pretreated with rHcL6 were incubated with normal rat IgG as the primary antibody (**B**). DAPI (blue) and Cy3-conjugated secondary antibodies (red) were utilized for double staining. Merge, overlap of Cy3 and DAPI channels. Scale bars, 10 μm.

A previous study found that the HcL6 protein is one of the ES components derived from HcESPs that interacts with host Th9 cells. To further validate the accuracy of this result, immunofluorescence staining assays were employed to confirm the in vitro interaction between the HcL6 protein and goat Th9 cells (Figure 2). The immunocytochemistry assays revealed intense red Cy3 fluorescence from the rHcL6-tagged cells in the rHcL6-treated Th9 sample. Moreover, neither the blank nor the negative control groups showed any red fluorescence. These findings offer further validation of the positive interaction between the HcL6 protein and host Th9 cells.

### rHcL6 induces a goat Th9 immune response

**Figure 3:**
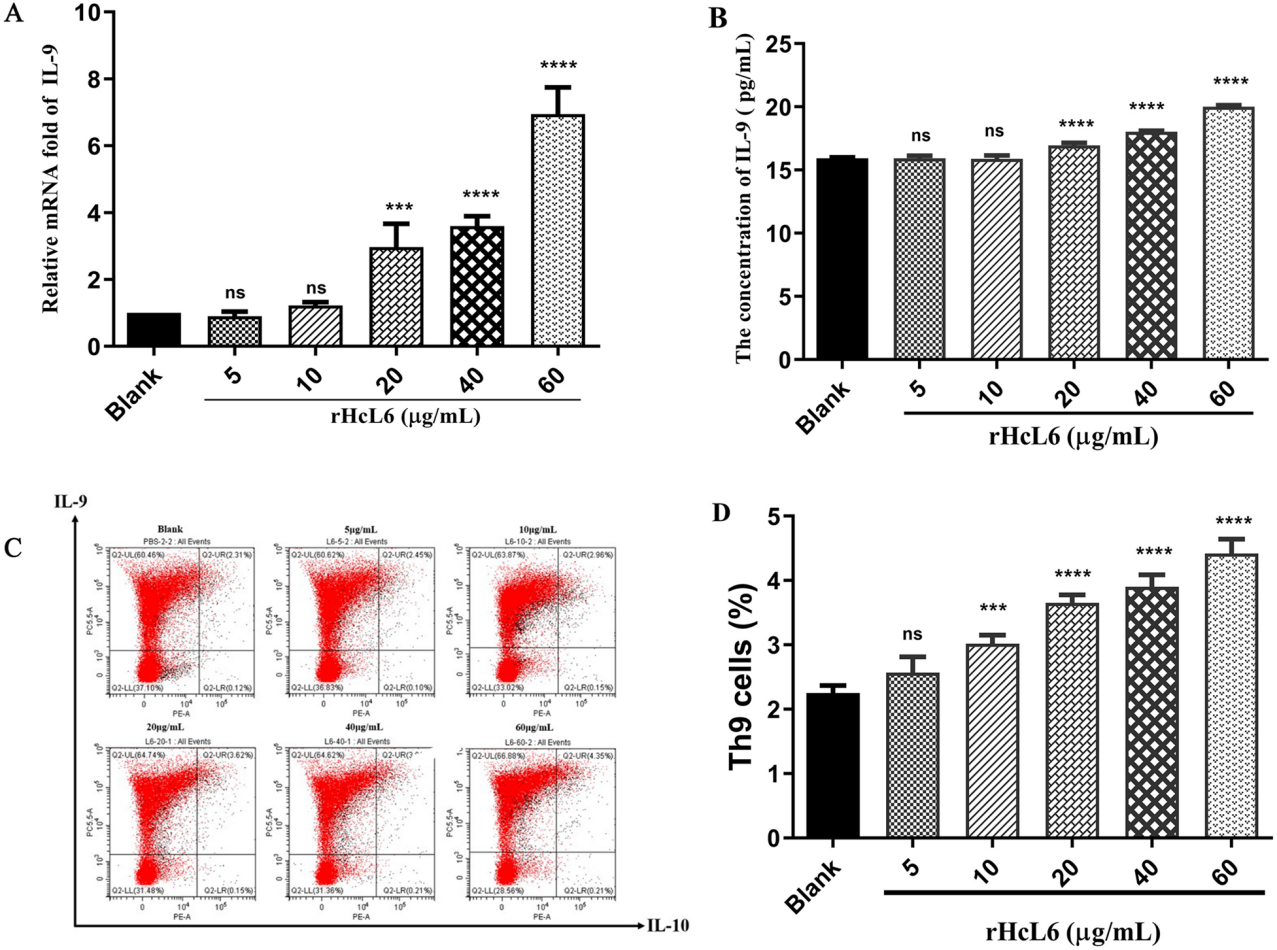
**rHcL6 induces a goat Th9 immune response**. Goat PBMCs were stimulated with different concentrations of rHcL6 (0, 5, 10, 20, 40 and 60 μg/mL). The significance level was set at ***p < 0.001, ****p < 0.0001, and “ns” as nonsignificant compared with the control (blank, 0 μg/mL). Data are representative of three independent experiments. **A**: Relative fold change in IL-9 transcription. **B**: IL-9 secretion profiles. The secretion of IL-9 in the culture supernatant was determined by ELISA. **C**: The effects of rHcL6 on Th9 cell proliferation. Th9 cells were detected by flow cytometry using typical intracellular cytokine antibodies (IL-9 and IL-10). **D**: Proportion of the Th9 cell population with different concentrations of rHcL6.

The populations of Th9 cells and the levels of IL-9 transcription and secretion were used to demonstrate whether rHcL6 could induce the goat Th9 immune response. qPCR assays were performed to assess IL-9 transcription levels after treatment with different concentrations of rHcL6. The relative mRNA fold changes in the groups treated with 20, 40 and 60 μg/mL rHcL6 increased to 2.97 (*p* < 0.001), 3.59 (*p* < 0.0001) and 6.94 (*p* < 0.0001), respectively, when compared with the blank control (PBS, fold = 1), while those treated with 5 μg/mL (fold = 0.90, *p* > 0.05) or 10 μg/mL (fold = 1.22, *p* > 0.05) did not show significant differences when compared with levels in the control group (Figure 3A).

To investigate the modulatory effects of rHcL6 on Th9 cells, IL-9 secretion was determined by ELISA. The results showed that goat Th9 cells exposed to rHcL6 stimuli altered IL-9 production profiles. IL-9 secretion was significantly promoted by treatment with more than 20 μg/mL rHcL6 (*p* < 0.0001) (Figure 3B). However, less than 20 μg/mL rHcL6 had no significant effects on the secretion of IL-9 compared with that in the control group.

To estimate the effects of rHcL6 on Th9 cells, goat PBMCs were incubated with different concentrations of rHcL6, and the population of Th9 cells was investigated. The flow cytometry results revealed that 60 μg/mL rHcL6 induced the highest production of Th9 cells, with a percentage of 4.35% (Figure 3C). Treatment with 5, 10, 20 and 40 μg/mL rHcL6 also promoted the generation of Th9 cells, with proportions of 2.45%, 2.96%, 3.62% and 3.81%, respectively (Figure 3C). The results showed that treatment with rHcL6 (10–60 μg/mL) significantly stimulated an increase in the number of Th9 cells compared with that in the control group (0 μg/mL, 2.31%) (Figure 3D).

### rHcL6 increased the expression of some key genes in Th9 immune response signaling pathways

**Figure 4:**
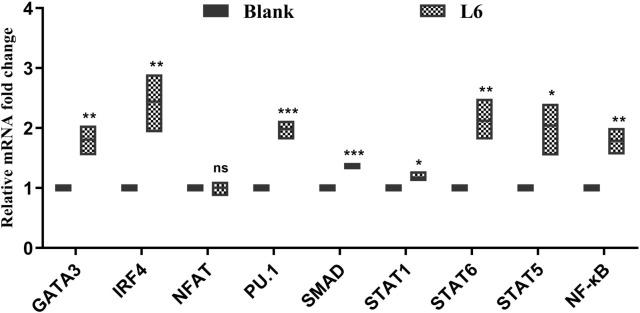
**Key genes involved in Th9 pathways increased by rHcL6 stimulation**. Relative fold changes in GATA3, IRF4, PU.1, SMAD, STAT1, STAT6, STAT5, NFAT and NF-ĸB transcription. qPCR was used to determine the transcription of these genes after goat PBMCs were incubated with 20 μg/mL rHcL6 for 24 h. The significance levels were set at *p < 0.05, **p < 0.01, ***p < 0.001, and “ns” as nonsignificant compared with the control (blank, 0 μg/mL). Data are representative of three independent experiments.

NF-κB, NFAT, STAT6, IRF4, STAT5, PU.1, SMAD, GATA3 and STAT1 are required for Th9 cell development. Real-time PCR analysis revealed that the mRNA transcripts of these genes were detectable. Increased expression levels were observed for GATA (fold = 1.81, *p* < 0.01), IRF4 (fold = 2.44, *p* < 0.01), PU.1 (fold = 1.98, *p* < 0.001), SMAD (fold = 1.37, *p* < 0.001), STAT1 (fold = 1.17, *p* < 0.05), STAT6 (fold = 2.12, *p* < 0.01), STAT5 (fold = 2.04, *p* < 0.05) and NF-ĸB (fold = 1.78, *p* < 0.01) compared to those in the blank control group (PBS, fold = 1). Nevertheless, NFAT exhibited no significant changes (1.01-fold, *p* > 0.05) compared with that in the blank control group (PBS, onefold) (Figure 4).

### Suppression of Th9 immune responses by knockdown of the STAT6 gene

**Figure 5:**
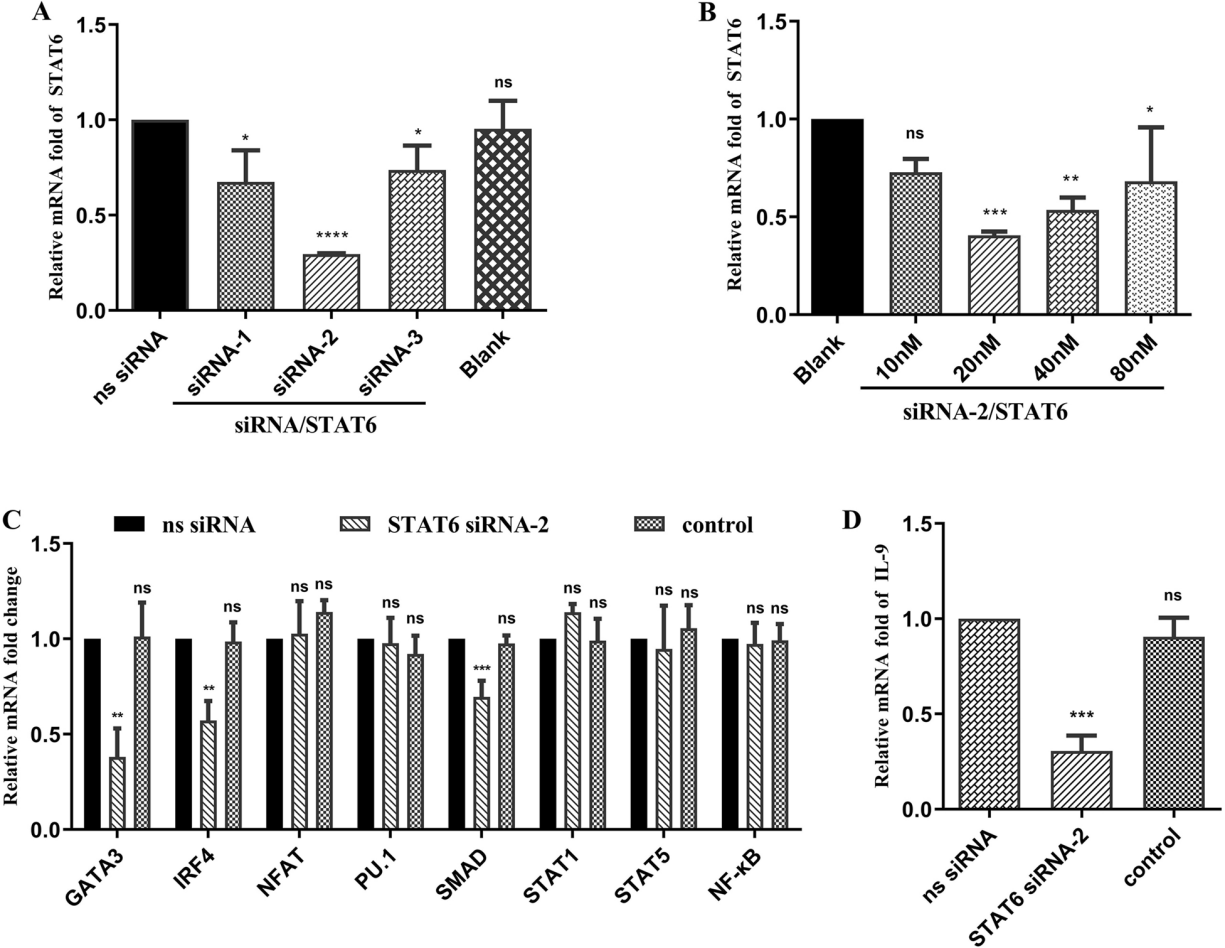
**Th9 immune response suppression following STAT6 gene knockdown**. **A**: Efficiency of STAT6 interference using different siRNAs. The transcription of STAT6 was detected using qPCR by introducing different siRNA/STAT6 sequences (siRNA-1, siRNA-2, and siRNA-3) and nonspecific (ns) siRNA using RNAiMAX Transfection Reagent for 48 h. Only RNAiMAX Transfection Reagent was added to the blank group. The significance levels were set as follows: *p < 0.05 and ****p < 0.0001, with “ns” denoting nonsignificance compared with the ns siRNA group. **B**: Efficiency of STAT6 interference with different concentrations of siRNA-2/STAT6. Goat peripheral blood mononuclear cells (PBMCs) were treated with different concentrations of siRNA-2/STAT6 (0, 10, 20, 40, and 80 µg/mL) using RNAiMAX Transfection Reagent for 48 h. The significance levels were defined as *p < 0.05, **p < 0.01, and ***p < 0.001, with “ns” representing nonsignificance compared with the control (blank, 0 nM). Subsequently, goat PBMCs were incubated with 20 µg/mL rHcL6 for 24 h after the addition of 20 nM siRNA-2/STAT6 and ns siRNA using RNAiMAX Transfection Reagent. The control group was also treated with 20 µg/mL rHcL6 for 24 h after the addition of RNAiMAX Transfection Reagent. The significance levels were set at **p < 0.01, and ***p < 0.001, with “ns” indicating nonsignificance compared with the ns siRNA group. Data are representative of three independent experiments. **C**: Relative fold changes in GATA3, IRF4, PU.1, SMAD, STAT1, STAT5, NFAT, and NF-ĸB transcription after STAT6 gene knockdown. **D**: Relative fold change in IL-9 transcription after STAT6 gene knockdown.

The above results show that HcL6 probably induces a Th9 immune response through stimulation of STAT6. Therefore, the STAT6 gene was knocked down to demonstrate its importance in the Th9 immune response. Three unique sequences of STAT6 siRNA were evaluated to determine the most effective sequence (Figure 5A). The optimal sequence, namely, STAT6 siRNA-2, significantly reduced STAT6 expression to 0.30 (*p* < 0.001). The introduction of increasing concentrations of STAT6 siRNA-2 (0, 10, 20, 40, and 80 nM) into peripheral blood mononuclear cells (PBMCs) led to a decrease in STAT6 transcription. The relative mRNA levels in the experimental groups were reduced to 0.72 (*p* > 0.05), 0.40 (*p* < 0.001), 0.53 (*p* < 0.01), and 0.68 (*p* < 0.05) at 10, 20, 40, and 80 nM, respectively, when compared to levels in the control (0 nM, fold = 1) (Figure 5B). Thus, 20 nM siRNA-2/STAT6 was identified as the optimal concentration. We examined the impact of small interfering RNA (siRNA) on the transcription of key genes in Th9 immune response signaling pathways. In comparison to those in the control (nonspecific siRNA), a decrease in expression levels was observed for GATA3 (*p* < 0.01), IRF4 (*p* < 0.01), and SMAD (*p* < 0.001) upon adding 20 nM siRNA-2/STAT6. However, NFAT, PU.1, STAT1, STAT5, and NF-κB did not show significant alterations compared to the control (Figure 5C). We also investigated the impact of siRNA on the transcription of IL-9. When 20 nM siRNA-2/STAT6 was added, the relative mRNA levels decreased to 0.31 (*p* < 0.001) compared to those in the control (nonspecific siRNA, fold = 1) (Figure 5D).

### Suppression of Th9 immune responses by knockdown of the NF-κB gene

**Figure 6:**
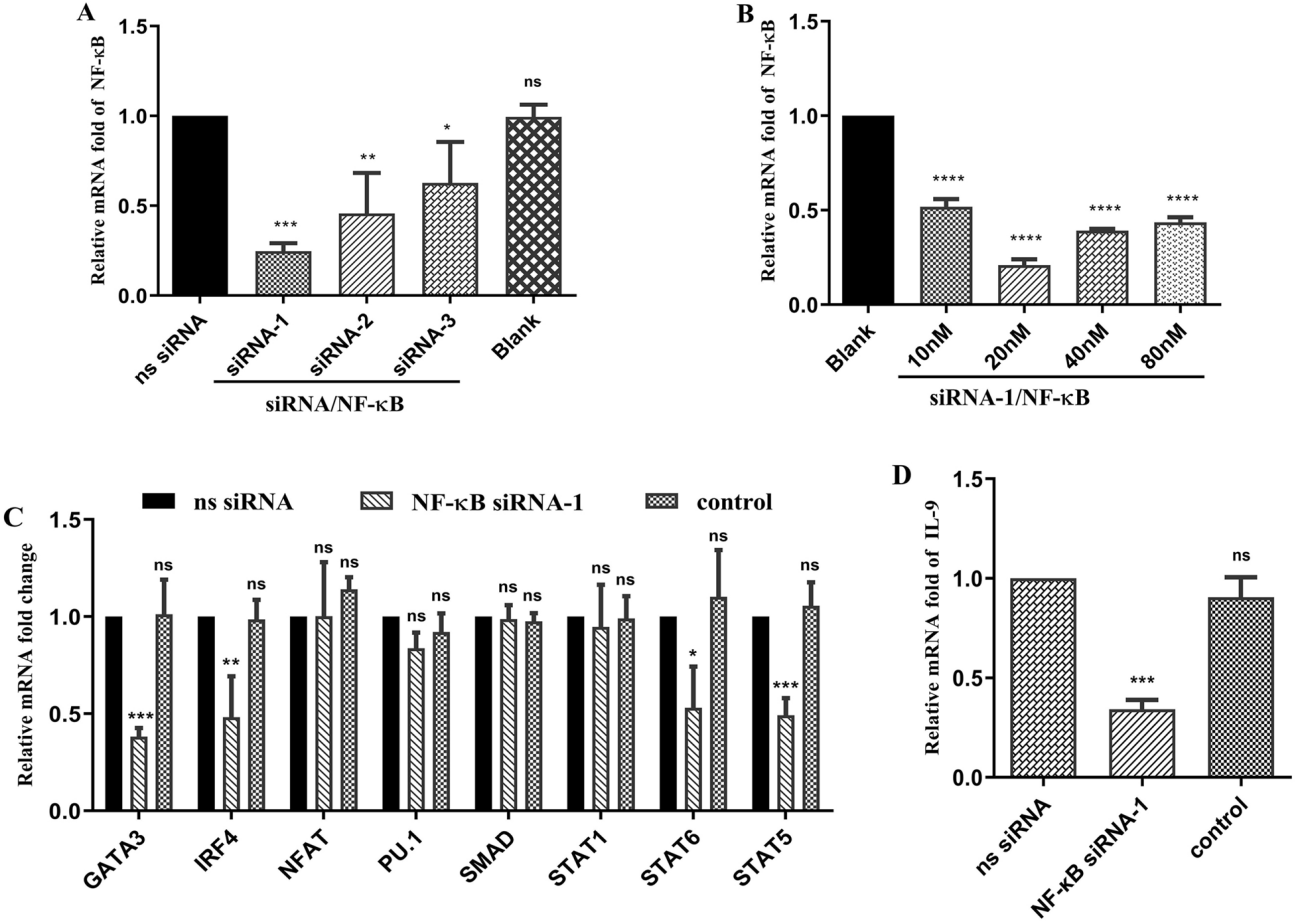
**Th9 immune response suppression following NF-κB gene knockdown**. **A**: Efficiency of NF-κB interference using different siRNAs. The transcription of NF-κB was detected using qPCR by introducing different siRNA/NF-κB sequences (siRNA-1, siRNA-2, and siRNA-3) and nonspecific (ns) siRNA using RNAiMAX Transfection Reagent for 48 h. Only RNAiMAX Transfection Reagent was added to the blank group. The significance levels were set as follows: *p < 0.05, **p < 0.01, and ***p < 0.001, with “ns” denoting nonsignificance compared with the ns siRNA group. **B**: Efficiency of NF-κB interference with different concentrations of siRNA-1/NF-κB. Goat peripheral blood mononuclear cells (PBMCs) were treated with different concentrations of siRNA-1/NF-κB (0, 10, 20, 40, and 80 µg/mL) using RNAiMAX Transfection Reagent for 48 h. The significance levels were defined as ****p < 0.0001, with “ns” representing nonsignificance compared with the control (blank, 0 nM). Subsequently, goat PBMCs were incubated with 20 µg/mL rHcL6 for 24 h after the addition of 20 nM siRNA-1/NF-κB and ns siRNA using RNAiMAX Transfection Reagent. The control group was also treated with 20 µg/mL rHcL6 for 24 h after the addition of RNAiMAX Transfection Reagent. The significance levels were set at *p < 0.05, **p < 0.01, and ***p < 0.001, with “ns” indicating nonsignificance compared with the ns siRNA group. Data are representative of three independent experiments. **C**: Relative fold changes in GATA3, IRF4, PU.1, SMAD, STAT1, STAT6, STAT5 and NFAT transcription after NF-κB gene knockdown. **D**: Relative fold change in IL-9 transcription after NF-κB gene knockdown.

To evaluate the role of NF-κB in the Th9 immune response, the transcription levels of IL-9 and other factors were observed after knockdown of the NF-κB gene. NF-κB siRNA-1 was the most appropriate sequence among the three single sequences of NF-κB siRNA (Figure 6A). It reduced the expression of NF-κB to 0.25 (*p* < 0.001). Increasing concentrations (0, 10, 20, 40 and 80 nM) of NF-κB siRNA-1 transfected into PBMCs resulted in a decrease in the transcription of NF-κB. The relative mRNA fold changes in the groups were reduced to 0.52 (*p* < 0.0001), 0.21 (*p* < 0.0001), 0.39 (*p* < 0.0001) and 0.43 (*p* < 0.0001) for NF-κB transcription at 10, 20, 40 and 80 nM, respectively, when compared with that in the control (0 nM, fold = 1) (Figure 6B). It was shown that 20 nM siRNA-1/NF-κB was optimal. We tested the impact of adding small interfering RNA (siRNA) against some key genes in the Th9 immune response signaling pathway. Decreased expression levels of GATA3 (*p* < 0.001), IRF4 (*p* < 0.01), STAT6 (*p* < 0.05) and STAT5 (*p* < 0.001) were observed after the addition of 20 nM siRNA-1/NF-ĸB compared to the control (ns siRNA). Nevertheless, NFAT, PU.1, SMAD and STAT1 had no significant effects compared with the control (ns siRNA) (Figure 6C). We tested the impact of adding small interfering RNA (siRNA) on the transcription of IL-9. The relative mRNA fold change in the group treated with 20 nM siRNA-1/NF-κB decreased to 0.34 (*p* < 0.001) when compared with that of the control (ns siRNA, fold = 1) (Figure 6D).

### Suppression of Th9 immune responses by knockdown of the PU.1 gene

**Figure 7:**
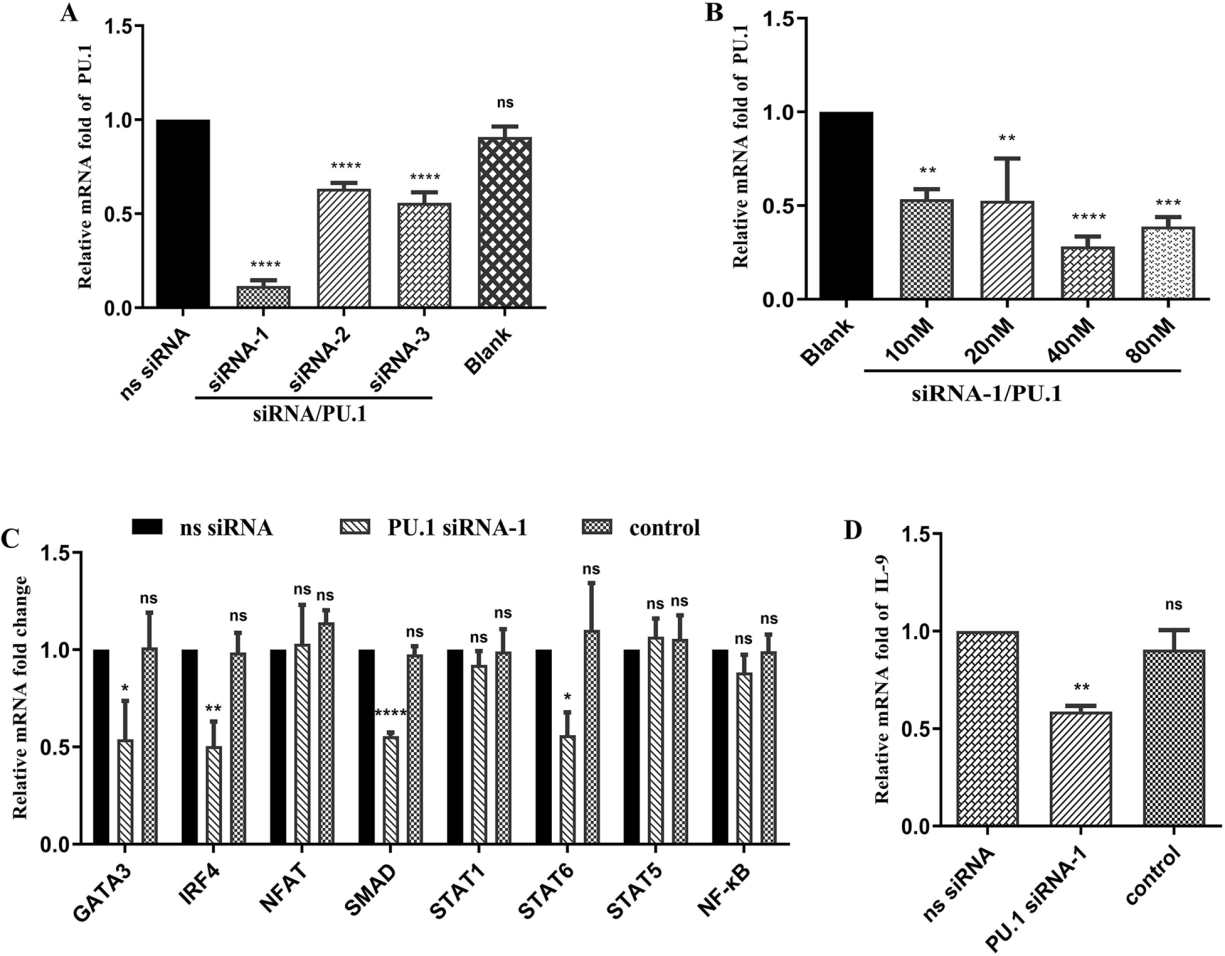
**Th9 immune response suppression following PU.1 gene knockdown**. **A**: Efficiency of PU.1 interference using different siRNAs. The transcription of PU.1 was detected using qPCR by introducing different siRNA/PU.1 sequences (siRNA-1, siRNA-2, and siRNA-3) and nonspecific (ns) siRNA using RNAiMAX Transfection Reagent for 48 h. Only RNAiMAX Transfection Reagent was added to the blank group. The significance levels were set as ****p < 0.0001, with “ns” denoting nonsignificance compared with the ns siRNA group. **B**: Efficiency of PU.1 interference with different concentrations of siRNA-1/PU.1. Goat peripheral blood mononuclear cells (PBMCs) were treated with different concentrations of siRNA-1/PU.1 (0, 10, 20, 40, and 80 µg/mL) using RNAiMAX Transfection Reagent for 48 h. The significance levels were defined as **p < 0.01, ***p < 0.001, and ****p < 0.0001, with “ns” representing nonsignificance compared with the control (blank, 0 nM). Subsequently, goat PBMCs were incubated with 20 µg/mL rHcL6 for 24 h after the addition of 40 nM siRNA-1/PU.1 and ns siRNA using RNAiMAX Transfection Reagent. The control group was also treated with 20 µg/mL rHcL6 for 24 h after the addition of RNAiMAX Transfection Reagent. The significance levels were set at *p < 0.05, **p < 0.01, and ****p < 0.0001, with “ns” indicating nonsignificance compared with the ns siRNA group. Data are representative of three independent experiments. **C**: Relative fold changes in GATA3, IRF4, SMAD, STAT1, STAT6, STAT5, NFAT and NF-ĸB transcription after PU.1 gene knockdown. **D**: Relative fold change in IL-9 transcription after PU.1 gene knockdown.

PU.1 also participates in the rHcL6-induced Th9 immune response. Transcription levels of IL-9 and other factors were assessed after knockdown of the PU.1 gene to evaluate the role of PU.1 in the Th9 immune response. PU.1 siRNA-1 was the optimal sequence among the three single sequences of PU.1 siRNA (Figure 7A). It reduced the expression of PU.1 to 0.20 (*p* < 0.0001). Increasing concentrations (0, 10, 20, 40 and 80 nM) of PU.1 siRNA-1 transfected into PBMCs resulted in a decrease in the transcription of PU.1. The relative mRNA fold changes in the groups were reduced to 0.53 (*p* < 0.01), 0.52 (*p* < 0.01), 0.28 (*p* < 0.0001) and 0.38 (*p* < 0.001) for PU.1 transcription at 10, 20, 40 and 80 nM, respectively, when compared with the level of the control (0 nM, fold = 1) (Figure 7B). It was shown that 40 nM siRNA-1/PU.1 was optimal. We tested the impact of adding small interfering RNA (siRNA) against some key genes in the Th9 immune response signaling pathway. Decreased expression levels after adding 40 nM siRNA-1/PU.1 were observed for GATA3 (*p* < 0.05), IRF4 (*p* < 0.01), SMAD (*p* < 0.0001) and STAT6 (*p* < 0.05) compared to that of the control (ns siRNA). Nevertheless, NFAT, STAT1 STAT5 and NF-κB had no significant effects compared with the control (ns siRNA) (Figure 7C). We tested the impact of adding small interfering RNA (siRNA) on the transcription of IL-9. The relative mRNA fold change in the group treated with 40 nM siRNA-1/PU.1 decreased to 0.59 (*p* < 0.01) when compared with that of the control (ns siRNA, fold = 1) (Figure 7D).

## Discussion

The excretory-secretory products (ESPs) released by parasitic helminths promote infection and modulate host immune responses [22–24]. It is imperative to understand the mechanisms of action of ESPs for the development of methods to prevent and treat nematode infections. The HcL6 protein is one of the ES components derived from HcESPs that interact with host Th9 cells. The HcL6 gene was successfully cloned, and the recombinant rHcL6 protein was obtained in the present study. Further studies should be conducted to determine whether the protein induces a Th9 immune response and its mechanism of action in modulating the Th9 immune response.

IL-9-producing Th9 cells play a role in enhancing immunity against parasites and cancer but are highly affected by allergic disease and colitis. The CD4 + T-cell subset can produce IL-9 alone or IL-9 in combination with IL-10 but not IL-4, IL-5 or other cytokines [25, 26]. Therefore, Th9 cells are described as IL-9 + and IL-10 + cells [27] and not Th1, Th2, and Th17 cells. Previous studies have reported that other cells, such as eosinophils and bone marrow-derived mucosal mast cells (BMMCs), can also produce IL-9. However, IL-9 is mainly produced by host Th9 cells. The characteristics of IL-9 and Th9 cells used in this study were used to induce a Th9 immune response. The results in the present study showed that HcL6 promoted the differentiation of Th9 cells and increased the transcription level and secretion of IL-9 in vitro. The in vitro results indicated that HcL6 induced a Th9 immune response.

Several cytokines promote Th9 cell development. In addition to TGF-β and IL-4, transcription factors such as NF-κB, NFAT, STAT6, IRF4, STAT5, PU.1, SMAD, GATA3 and STAT1 are required for Th9 cell development [6–10, 28, 29]. The real-time PCR results showed that coincubation of HcL6 with PBMCs significantly upregulated the transcription of GATA3, IRF4, PU.1, SMAD, STAT1, STAT6, STAT5, and NF-κB. These results show that HcL6 promotes the differentiation of Th9 cells and the expression of IL-9 probably through stimulation of these transcription factors, indicating a complex signal transduction network in the differentiation of Th9 cells.

The Janus kinases JAK1 and JAK3 are activated when IL-4 induces IL-4Ra. Subsequently, STAT6 is recruited and phosphorylated, and genes that regulate Th2 cell development are expressed [30]. Notably, phosphorylated STAT6 is essential for IL-9 production because IL-4 plays a key role in Th9 cell development. STAT6-deficient CD4 + T cells do not produce IL-9 when cultured under Th9 polarized conditions [7, 9]. After activation, phosphorylated STAT6 promotes the transcription of Gata3 and IRF4, which are essential for the production of Th2 and Th9 cells [8, 31]. In this study, the transcription of GATA3 and IRF4 was downregulated after the addition of small interfering RNA (siRNA) against STAT6. This is consistent with findings from previous research.

TGF-β-induced expression of PU.1 is required for IL-9 production [32]. PU.1 suppresses the Th2 phenotype and enhances the Th9 phenotype by acting as a bridge between Th2 cells and Th9 cells. PU.1 modulates Th9 production by directly binding to the Il9 locus and recruiting Gcn5 [Kat2a, K(lysine) acetyltransferase 2A] and PCAF [Kat2b, K(lysine) acetyltransferase 2B] [32, 33], resulting in the formation of open chromatin at the Il9 locus and promoting the binding of transcription factors such as IRF4 for Il9 gene expression. PU.1-deficient Th9 cultures exhibit decreased acetylation of total histone H3, H3K9/18, H3K14, H4K5, H4K8 and H4K16 at the Il9 locus. Therefore, this finding indicates that the PU.1/Gcn5 complex is essential for the development of Th9 cells. This result was also confirmed in the present study, wherein the addition of small interfering RNA (siRNA) against PU.1 decreased the transcription levels of STAT6, GATA3, IRF4 and SMAD.

RelA, c-Rel, RelB, NF-κB1 (p50 and its precursor p105) and NF-κB2 (p52 and its precursor p100) are members of the mammalian Rel/NF-κB transcription factor family. These proteins play important roles in the immune system by regulating the development, survival, immune responses, and malignant transformation of lymphocytes and lymphoid organs. The p50-RelA and p52-RelB proteins are implicated in the NF-κB signaling pathway [34]. The NF-κB-mediated pathway is involved in the induction of IL-9. NF-κB and nuclear factor of activated T cells (NFAT1) are required for TCR-induced IL-9 production by Th9 cells [35]. Binding of NFAT1 and NF-κB (p65) to the IL-9 promoter was observed in vivo. NFAT1 binding at the promoter site was followed by the presence of a transcriptionally active chromatin configuration at the promoter site of the IL-9 gene, which was activated by the binding of NF-κB (p65). In NFAT1-deficient mice, IL-9 expression is significantly downregulated due to histone modification and altered chromatin structure partly caused by recruitment of HAT p300 to the Il9 promoter. Furthermore, knockdown of NF-κB (p65) decreases IL-9 expression levels [35].

**Figure 8:**
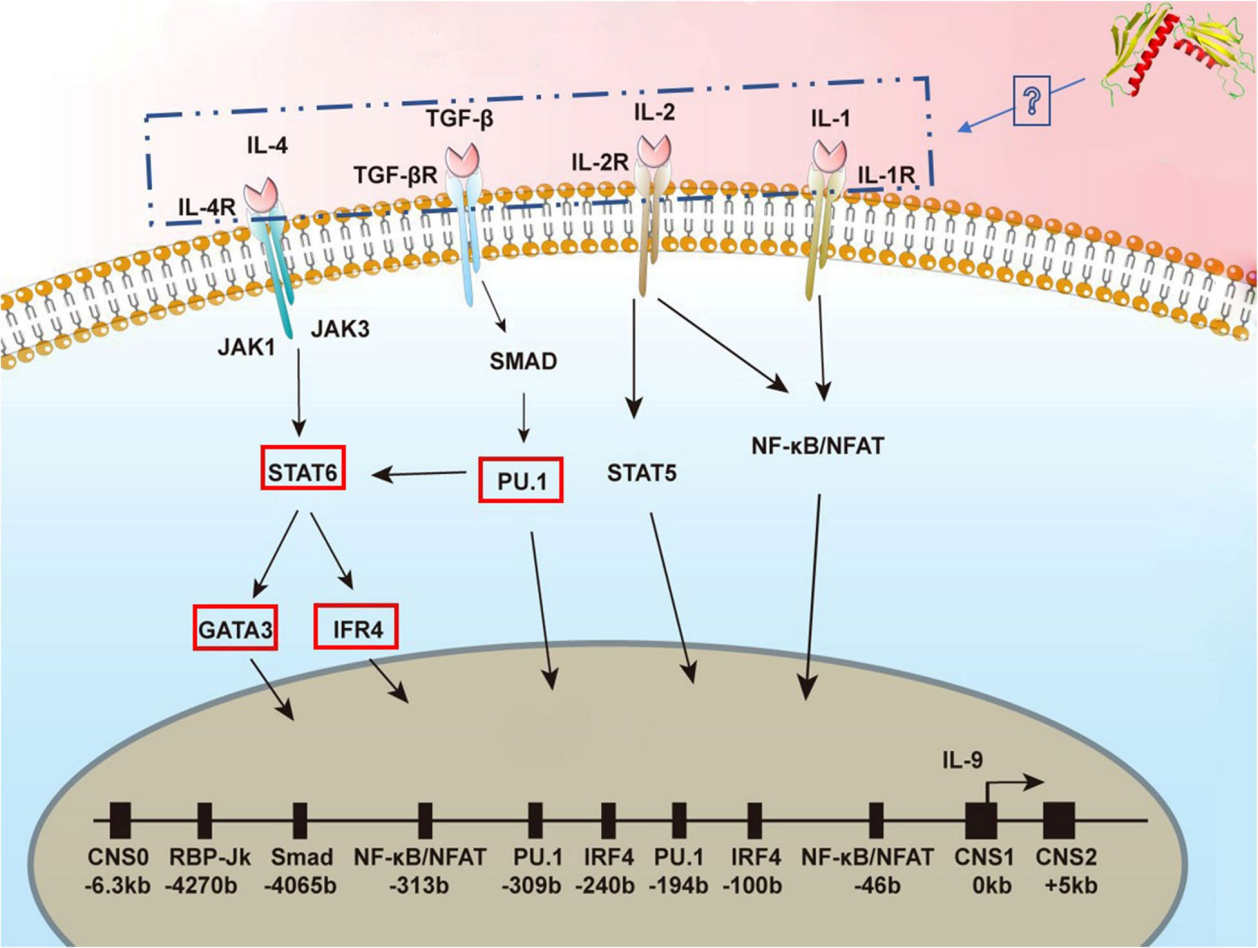
**Activation of the Th9 immune response by HcL6 through the IL-4/STAT6/IRF4-GATA3 and TGF-β/PU.1 signaling pathways.** The coincubation of HcL6 with peripheral blood mononuclear cells (PBMCs) significantly upregulated the transcription of GATA3, IRF4, PU.1, and STAT6. Knockdown of the PU.1 and STAT6 genes suppressed the expression of STAT6, GATA3, and IRF4.

In this study, we employed small interfering RNA (siRNA) to investigate the transcription levels of pivotal genes in Th9 immune response signaling pathways associated with STAT6/NF-κB/PU.1 synthesis. The outcomes showed that following treatment with siRNA, the expression levels of GATA3, IRF4, SMAD, STAT6, and PU.1 were downregulated. These findings suggest that HcL6 may stimulate the Th9 immune response via activation of the IL-4/STAT6/IRF4-GATA3 and TGF-β/PU.1 signaling-mediated transduction pathways (Figure 8).

### Supplementary Information

**Additional file 1: Sequence of the HcL6 gene.** The complete coding sequence of the HcL6 gene (567 bp).

## Data Availability

The datasets supporting the conclusions of this article are included in Additional file 1.

## Abbreviations

ESPs: excretory and secretory proteins
HcL6: *H. contortus* Ribosomal protein L6 domain DE-containing protein
rHcL6: recombinant HcL6
PBS: phosphate-buffered saline
IL: interleukin
PBMC: peripheral blood mononuclear cell
