# Supplementary material for: Haemonchus contortus HcL6 promoted the Th9 immune response in goat PBMCs by activating the STAT6/PU.1/NF-κB pathway
Source: Vet Res. 2023 Sep 22;54:80. doi: 10.1186/s13567-023-01214-5 (PMC10517550; doi:10.1186/s13567-023-01214-5)
Supplement: Supplementary file 1 — Additional file 1: Sequence of the HcL6 gene. The complete coding sequence of the HcL6 gene (567 bp). [file 13567_2023_1214_MOESM1_ESM.docx]

**Sequence：**ATGAAGCTGGTCGAGTCCAATGATACCGTAGATTTCCCTGATGGTGTGACGTTCACCGTCAAAAACCGCGTAGTTCACGTTACCGGACCTCGCGGAACACTAAAACGTGACTTCCGTCACCTTCACATGGAGATGGAACGTGTCGGGAAGAACCAGCTGCGTGTACGCAAGTGGTTCGGAGTTCGCAAAGAGATCGCTGCCATTCGAACAGTGTGCTCACACATCCAGAACATGATCAAGGGTGTCACTAAGGGTTTCCGCTACAAGATGCGATCCGTATACGCCCATTTCCCCATCAACGTCACTCTTCAAGATGGTGGAAGAACTGTTGAGATTCGTAACTTCCTCGGAGAAAAGATTGTTCGTCGTGTGCCCCTTCCGGATGGTGTCACTGCCACGTTGTCCACTTCACAAAAGGACGAGCTCATCATTGAAGGAAACGATATTCAGCTCGTATCTCAAGCTGCCGCTCGTATCCAGCAGTCTACGTCTGTTAAAGAAAAGGATATCCGTAAGTTCCTTGA

TGGAATCTACGTGTCAGAGAAAACTACTATTGTTCAAGACTGA
